# In Silico Screening of Synthetic and Natural Compounds to Inhibit the Binding Capacity of Heavy Metal Compounds against EGFR Protein of Lung Cancer

**DOI:** 10.1155/2022/2941962

**Published:** 2022-05-14

**Authors:** Zainab Ayaz, Bibi Zainab, Umer Rashid, Noura M. Darwish, Mansour K. Gatasheh, Arshad Mehmood Abbasi

**Affiliations:** ^1^Department of Environmental Sciences, COMSATS University Islamabad, Abbottabad Campus 22060, Pakistan; ^2^Department of Chemistry, COMSATS University Islamabad, Abbottabad Campus 22060, Pakistan; ^3^Faculty of Science Ain Shams University, Biochemistry Department, Abbasaya, P.O. Box. 11566, Cario, Egypt; ^4^Ministry of Health Laboratories, Tanta, Egypt; ^5^Department of Biochemistry, College of Science, King Saud University, Riyadh 11451, Saudi Arabia; ^6^University of Gastronomic Sciences, Piazza Vittorio Emanuele II, 9, 12042 Pollenzo, Italy

## Abstract

Inorganic pollutant, specifically heavy metals' contamination, is a significant matter of concern and is one of the key contributors in various health disorders including cancer. However, the interaction of heavy metals (HMs) with lung cancer has rarely been explored yet. Therefore, the present study was intended with the aim to identify the interactions of HMs with the target protein “epidermal growth factor receptor (EGFR)” of lung cancer and explore potential drug candidates, which could inhibit the active site of EGFR against HM exposure. The molecular operating environment (MOE) tool was used to study the interactions of HMs with EGFR protein. The drug-drug interaction (DDI) network approach was used to identify the potential drug candidates, which were further confirmed and compared with the commercial medicines/control group. Various compounds of twenty-three HMs were docked with EGFR protein. Out of which tinidazole, thallium bromodimethyl, and silver acetate (Sn, Ti, and Ag compounds) showed strong interactions with EGFR based on lowest-scoring values (-20.42, -7.86, and -7.74 kcal/mol, respectively). Among 1280 collected drug candidates, three synthetic compounds viz., ZINC00602803, ZINC00602685, and ZINC06718468 and three natural compounds (berberine chloride, transresveratrol, and ellagic acid) depicted strong binding capacity with EGFR. Specifically, the scoring value of ZINC00602803 (-30.99 kcal/mol) was even lowest than standard lung cancer drugs (afatinib, erlotinib, and gefitinib). Our findings revealed that both natural and synthetic compounds having strong associations with EGFR protein could be potential candidates to inhibit the interaction between HMs and lung cancer protein and can also be used as an alternative for the prevention and treatment of lung cancer. However, *in vitro* and *in vivo* studies should be conducted to validate the aforementioned natural and synthetic compounds.

## 1. Introduction

Natural, geogenic, lithogenic, and anthropogenic activities are major contributors to environmental contamination [1, 2]. Various organic and inorganic toxins enter the human body through ingestion, inhalation, and dermal contact from contaminated water, soil, air, and food and cause various health effects. Potential toxic substances such as heavy metals (HMs) and persistent organic pollutants (POPs) have received global paramount attention by scientists due to their lethal nature [1, 3]. Both natural (weathering of rocks and vulcanization) and anthropogenic activities viz., industrialization, urbanization, automobiles, and extensive use of agrochemicals are major causes of food chain contamination [4, 5]. Various epidemiological studies have illustrated that consumption of contaminated foods, specifically containing HMs, is one of the major threats to human health. Such as intake of arsenic, cadmium, chromium, lead, mercury, and tin via ingestion of contaminated foods causes various types of cancers (lung, bladder, breast, esophagus, stomach, intestines, prostate, and skin cancer) in humans [6–8].

Based on the recent advancements, computer-based approaches satisfy environmental scientists/regulators in estimating the properties of compounds, analyzing their fate-determining processes, and feasibly predicting their results [9]. Environmental informatics (EI), a computational approach [10], is actively addressing the daunting issues and bridges the gap between computer science and environmental sciences [11]. Awareness of the desired orientation in molecular docking can be used to determine the strength or affinity of contact between two molecules [12].

Lung cancer is the most common cancer in men and the fourth most common cancer in women [13, 14]. International Agency for Research on Cancer (IARC) has estimated that approximately 2.09 million cases of lung cancer are diagnosed every year along with 1.76 million deaths [14]. However, there is a scarcity of data reflecting the true incidence and mortality of lung cancer in Pakistan. Global Cancer Incidence, Mortality, and Prevalence (GLOBOCON, 2012) placed lung cancer as Pakistan's third most common cancer, while Pakistan Health Research Council (PHRC, 2016), data revealed that lung cancer in Pakistan is the 10th most common cancer [15]. It has been assumed that industrialization, urbanization, and agricultural practices cause overexposure and long-term bioaccumulation of different heavy metals in the environment and food products, which increase the incidence of lung cancer [8, 16].

Approximately, 80-85% of lung cancers are non-small-cell lung cancer (NSCLC) [17]. However, in association with some lung cancers, mutations in epidermal growth factor receptor (EGFR) have been reported [18]. The EGFR is a transmembrane receptor tyrosine kinase protein in some normal epithelial, mesenchymal, and neurogenic tissue that regulates signaling pathways but its overexpression has been reported in the pathogenesis of many human malignancies, including NSCLC [18]. Half of the newly diagnosed patients with NSCLC have progressive disease, which offers a poor prognosis due to the drug resistance of EGFR protein (landmark target of NSCLC). And effectiveness of the standard drugs such as erlotinib, gefitinib, and afatinib is limited [17]. To resolve acquired EGFR resistance, various strategies have been explored but still, monotherapy in the first line is needed to be developed [19]. Though it is well established that HMs are contributing to various types of cancer, their association with EGFR protein is not clearly understood yet. Therefore, present findings could contribute to understanding the mechanism of molecular interactions of various inorganic toxins, specifically HMs and their role in lung cancer and the application of alternative compounds to treat this lethal health disorder. It has been reported that human exposure to certain HMs present in his surrounding environment increases the risk of lung cancer; especially, Cd, Cr, Ni, and Pb contents were found significantly higher in the urine of lung cancer patients compared to noncancer controls [20]. Various studies are in the view that HMs contribute in lung cancer. Such as arsenic (As) and beryllium (Be) compounds depicted significant association with lung cancer both *in vitro* and *in vivo* [21, 22]. The International Agency for Research on Cancer and the US National Toxicology Program have classified cadmium (Cd) compounds as human carcinogens based on strong associations between occupational Cd exposure and lung cancer in humans [23], because Cd causes inflammation in human lungs via increased oxidative stress, resulting in tissue destruction, obstructive lung function, and cancer. However, there is a contradiction in the carcinogenic effect of iron (Fe), specifically its role in lung cancer [24]. In this context, the present study was intended with the aim (i) to study molecular interactions of various heavy metals' compounds with lung cancer protein “EGFR” using *in silico* approach and (ii) to explore the potential drug candidates from a database of synthetic and natural compounds to inhibit interactions between HMs and EGFR.

## 2. Materials and Methods

### 2.1. Disease Selection and Identification of Gene

Direct or indirect exposure to inorganic toxins, like HMs, could contribute to various types of cancer [25], which have been predicted to be the most significant threat to rising life expectancy in every nation of the world [26]. In this study, NSCLC, a common malignant form of lung cancer, was selected which is one of the most lethal type of cancer [14]. The selection and identification of mutated gene of lung cancer were based on previous literature [19] and were also subsequently confirmed using the online database GeneCards **(**https://www.genecards.org/**).** The GeneCards is an extensive, integrated, annotative, and sophisticated search engine [27]. Finally, EGFR gene was selected as a target based on its highest-scoring value, which was approximately 21.25 as given in Table 1.

### 2.2. Protein Selection and Preparation

The target protein was selected based on identified gene, and its structure was downloaded through Research Collaborator for Structural Bioinformatics Protein Data Bank (RCSB PDB) database **(**https://www.rcsb.org/**)**, which provides structural data information of biological molecules [28]. Protein preparation was carried out using the MOE tool, which is a widely used program for chemical computing, molecular modeling, and other scientific applications [29]. Open Sequence Editor module was used to delete the nondesired chains and residues, followed by the addition of hydrogen bonds, while the energy of the protein molecule was minimized using the energy minimization algorithm. Energy minimization was settled when the root mean square gradient reaches less than 0.05.

### 2.3. Validation

To validate whether our approach can distinguish between active and inactive compounds, a virtual screen (VS) experiment was performed using actives (843 EGFR inhibitors, i.e., binders) as positive control and decoys (18000 compounds, i.e., nonbinders) as negative datasets obtained from the Database of Useful Decoys: Enhanced (DUD-E). All the dataset compounds were docked into the binding site of ER*α* (PDB ID: 6DUK).

### 2.4. Toxicity Prediction of Heavy Metals and Ligand Preparation

Data on various heavy metals were collected through literature [7] and online database PubChem (https://pubchem.ncbi.nlm.nih.gov/) as reported earlier [25]. In addition, admetSAR, a web server, was used to predict the chemical toxicity of the heavy metals [30], on humans, plants, animals, or the environment [31]. Ligand identification by any biomolecule depends on its three-dimensional orientation and electrostatic interactions [32]. To find the correct conformations, ligands were prepared through MOE tool, in which ground state geometries of the ligands were optimized through energy minimization.

### 2.5. Molecular Docking

Molecular docking was employed to explore the possible binding mode between a small molecule (ligand) and the target protein or receptor [33]. MOE was used for molecular docking [34], and calculations were carried out based on *S*-value and RMSD value. Before docking the database, the docking protocol was validated by using the redock method, and the cocrystallized ligand was redocked into the binding site of 6duk, and root mean square deviation (RMSD) was computed. The quality of docking accuracy/docking pose was assessed with the following RMSD values range: ≤1.10 = good pose, <1.11-1.90 = close pose (bold), and ≥2.00 bad pose (bold-italic) as mentioned in Table 2. Docking results were visualized and interpreted using 2D and 3D structures through Discovery Studio Visualizer.

### 2.6. Collection and Mining of Drug Candidates

ZINC database (https://zinc.docking.org/) supports virtual screening, ligand discovery, pharmacophore screens, benchmarking, and force field advancement [35] and was used to collect drug candidates (synthetic and natural) along with their structures and chemical properties such as “Zinc ID or drug ID, LogP, molecular weight, hydrogen bond donors (HBD), Hydrogen bond acceptors (HBA), rotatable bonds, non-polar dissociation, and polar dissociation.” In the process of data mining, the processed data from multiple perspectives is summarized into valuable information that can be used to raise revenue, reduce costs, or maybe both [36]. Lipinski rule of five was used to extract data, and according to the rule, value for hydrogen bond donor, hydrogen bond acceptors, segment coefficient log *P* esteem, and several rotatable bonds should be less than 5, 10, 5, and 10, respectively, and subatomic weight should also be less than 500 g/mol [37]. Therefore, drug candidates that comply with the Lipinski rule were selected.

### 2.7. Clustering of Screened Drug Candidates and DDI Network Generation

Clustering of drug candidates was accomplished through the Weka tool by the “simple K means clustering” method. In this method data set (*x*1, *x*2, *x*3 ⋯ ⋯.*xn*) was classified into *K* clusters according to their properties [38]. Drug-drug interaction networks facilitate in the identification of clear correlations of drug candidates within each cluster that supports the identification of strongly interacted drugs. Gephi tool was used in the generation of DDI networks, which is an open-access platform for importing, visualizing, spatializing, filtering, manipulating, and exporting all kinds of networks [39].

### 2.8. Validation of Drug Candidates

Only those drug candidates which have higher modularity values in a strong DDI network were selected, and their activity was further confirmed through docking. The drug candidates were validated through molecular docking that confirms the binding activity of drug candidates to the active site of EGFR protein. And compounds having the highest scoring value were recommended to be used exclusively or synergistically to attain optimal efficiency against lung cancer.

## 3. Results and Discussion

### 3.1. Selection of EGFR Protein's ID

**Figure 1:**
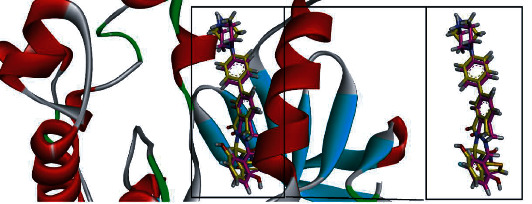
Demonstration of redocked cocrystallized ligand on experimental ligand.

The EGFR protein's ID was selected using a cross-docking approach.  Figure 1 illustrates the superposed diagram of the redocked ligand on the experimental ligand. Three-dimensional structures of three EGFR proteins were retrieved from PDB. For every available structure, each native ligand was docked. The results of cross-docking as mentioned in Table 2 indicate that docking simulations carried out on 3D structures in complex with different ligands had only about 44% of chance of reliable pose. Based upon cross-docking results for further studies, we used PDB ID “6DUK.”

### 3.2. Interaction of HMs with EGFR Protein

It is well established that heavy metals are suspected to enhance the ratio of different types of cancer in humans [40], including lung cancer. EGFR protein, having protein ID “6DUK”, one of the landmarks for lungs' cancer therapy was prepared for docking to evaluate the interactions with heavy metals and screened drug candidates. Oral toxicity of screened heavy metals (*n* = 23) was anticipated through admetSAR along with molecular weight, water solubility, and signal. As shown in Table 3, most of the HMs were lying in toxicity class 3. The molecular weight of screened HMs was ranged between 50 and 238 mg/mol, while their water solubility was between 0.03 and 11.3 mol/L.

According to the United States Environmental Protection Agency (USEPA) and the International Agency for Research on Cancer (IARC), various epidemiological studies have reported that As, Cd, Cr, Hg, Ni, and Pb are either classified as “known” or “probable” human carcinogens [41]. However, our findings revealed that tinidazole, thallium bromodimethyl, and silver acetate compounds of three heavy metals viz., tin (Sn), thallium (Ti), and silver (Ag) have strong associations with EGFR protein based on lowest-scoring values (-20.42, -7.86, and -7.74 kcal/mol, respectively) as demonstrated in Table 4.

Various factors including solubility, the ability of a metal to bind at active sites of proteins, and the degree to which the metal complexes are sequestered, metabolized, and excreted may affect the metal's ability to cause toxic effects [42]. Moreover, when metal is introduced into the body by the oral or dietary route, the liver substantially decreases its bioavailability to 90% through excretion. While, remaining amount, which is not disposed of, interacts with proteins by reacting to certain chemical groups in the protein's structure and forms a metal-protein complex. In the case of excessive doses, the removal pathways are saturated, and tissue deposition is increased. This facilitates the formation of such complexes that cause various adverse effects including cancer [43].

Different natural and anthropogenic activities involve in the contamination of HMs, specifically Sn, Tl, and Ag in the food chain. Sn compounds (organic and inorganic) are used in toothpaste, perfumes, soaps, coloring agents, food additives, and dyes, from where they enter into the human body through various routes, i.e., air, water, soil, and food. The provisional tolerable daily intake (PTDI) for tin is 14 mg/kg body weight, and recommended maximum permissible levels of tin in food are typically 150 mg/kg for canned beverages [44]. Coal-burning and smelting are primary sources of Tl contamination specifically, in the vicinity of industrial zones, elevated levels of Tl contaminate vegetables, fruits, and tissues of farm animals. The admissible limit of Tl in food is 0.1 mg/g while its oral reference dose is 0.056 mg/day/person. The toxicity of thallium-based compounds is mainly due to the similarity between thallium and potassium ions, and thallium interference creates disorder in potassium-associated metabolic processes [45]. Ag is used as a food additive and has also been used for surgical prostheses and splints, fungicides, and coinage [46]. But due to its adverse health effects, OSHA and the National Institute for Occupational Safety and Health (NIOSH) prescribed the permissible limit < 0.01 to 2.6 *μ*g/kg for all forms of silver [42].

### 3.3. Collection and Clustering of Drug Candidates

**Figure 2:**
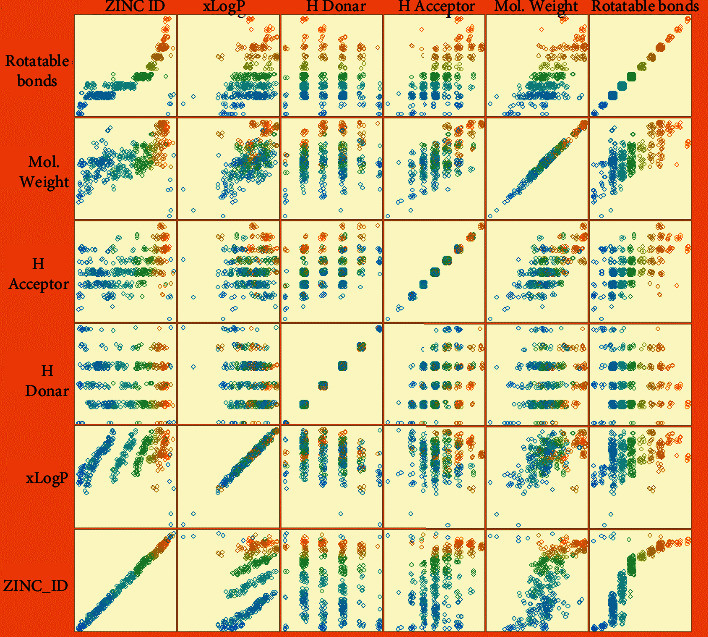
Plot matrix representation of drug candidates along with their attributes.

The retrieved data set of 1280 compounds from the ZINC database were filtered, and 1073 compounds were selected for further analysis based on Lipinski rule of five. Weka tool was used for clustering the data set of 1073 compounds. In total, eight clusters were made using the *K*-means algorithm, because of its efficacy in terms of execution time and implementation. In each cluster (Figure 2), drug candidates having similar properties are represented in bands according to their *x*- and *y*-axis properties. Majority of the drug candidates have an xLog*P* value between 1.17 and 4.98 and H-donor value between 0 and 2.5. However, maximum drug candidates having H acceptor value ranged from 4 to 5 and vice versa.

### 3.4. Drug-Drug Interaction (DDI) Networks and Their Statistics

**Figure 3:**
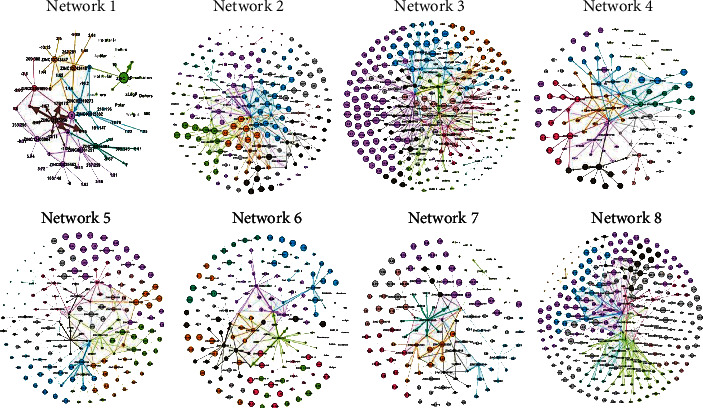
Drug-drug interaction networks of the entire collected data set of drug candidates.

To overcome the problem of large and complex data representation, the Gephi tool (0.9.1) which provides a platform for complex network visualization, analysis, good repositioning hints, and properties prediction was used [47]. Fruchterman rein gold parameters were used to generate DDI networks and to organize the random network for visualization and analysis. The repulsion strength of the modules in the Force Atlas was set to be 10,000 for the appropriate display of the network. Based on clustered data, eight DDI networks were generated based on modularity, path lengths, average degree, average weighted degree, degree distribution, and graph density (Figure 3). In each network, nodes represent the drug candidates while edges show the interactions among them. The sizes of the nodes vary due to the difference in their strength within a network while random colors were selected as a community identifier. Drug-drug interaction networks having smaller and larger sizes of nodes and edges represent the strength and partition of the communities within the network.

### 3.5. Statistics of Drug-Drug Interaction (DDI) Networks

Statistical parameters were calculated for each network. Average degree, average weighted degree, network diameter, graph density, modularity, average path length, number of nodes, and edges were considered for analysis (Table 5). In network analysis, community detection is of central importance. The modularity module was used for the study and detection of communities in a network. Based on modularity class, distant colors were assigned to nodes and edges. The modularity value of 0.4 or greater is generally considered meaningful for a network [48].

**Figure 4:**
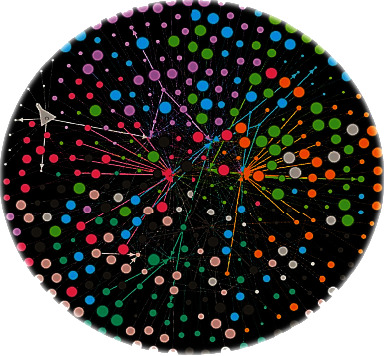
Final drug-drug interaction network of drug candidates having highest potential in the entire dataset.

In all networks, modularity values were mostly greater than 0.4. Comparatively, network 5 had the highest modularity value (0.629), while network 4 exhibited the lowest modularity value of 0.579. However, other parameters support in identifying the strongly contacted IDs of the networks. The first network comprises 72 nodes and 85 edges; the second and third networks have 207 nodes-300 edges and 276 nodes-386 edges, respectively. The fourth network has 3, 123 nodes and 163 edges, while 169 nodes and 215 edges were noted in the fifth network. There were 169 nodes and 218 edges in network six, 131 nodes and 172 edges in the seventh network, and 287 nodes and 403 edges were noted in network eight (Table 5). A final strong DDI network was generated using the drug candidates having higher modularity values as shown in Figure 4, which has 415 nodes and 740 edges. For partitioning the communities within the network, the modularity class was used. To analyze the final DDI network, the same parameters were applied as mentioned in Table 5. The modularity value of the final DDI network was 0.518, which means the entities/drug candidates of the network are significant.

### 3.6. Validation of Strongly Interacted Drugs

To examine the interaction of identified drug candidates against EGFR protein, molecular docking was performed using MOE, which is used in the screening of suitable ligand that fits both energetically and geometrically in the active site of targeted protein [49]. As the active site or binding cavity enables the protein to get attached to other macro or micro molecules [50]. Moreover, based on modularity values, out of 158 compounds, 55 were collected from the final DDI network. Selected drug candidates (*n* = 55) were docked with target protein, and their binding affinities were evaluated.

**Figure 5:**
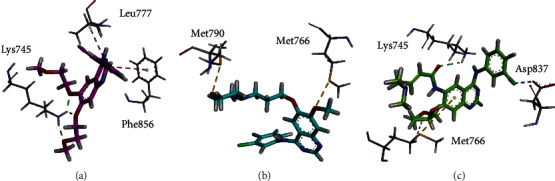
3D interactions of (a) erlotinib, (b) gefitinib, and (c) afatinib with binding site of target protein.

Three lung cancer drugs, i.e., erlotinib, gefitinib, and afatinib, were used as a positive control. As shown in Table 6, relatively, afatinib had the lowest-scoring value (-29.99 kcal/mol), followed by erlotinib and gefitinib (-29.49 and -29.33 kcal/mol, respectively). These drugs were docked with active sites of EGFR protein (Figures 5(a)–5(c)). Erlotinib drug exhibited pi-pi T-shaped and conventional hydrogen bond interactions with amino acids or active residues (Lys745, Leu777, Phe856) of target protein (Figure 5(a)); gefitinib showed sulfur-X bonds with active residues (Met790, Met766) of the target protein (Figure 5(b)), and afatinib had conventional hydrogen bond, halogen, pi-sulfur, and pi-donor hydrogen bond interactions with Lys745, Asp837, and Met766 active residues of target protein (Figure 5(c)).

**Figure 6:**
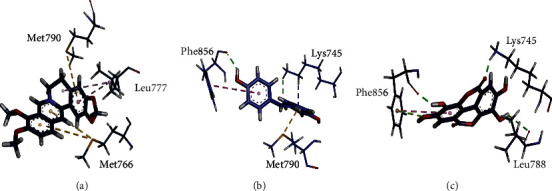
3D interactions of natural compounds (a) berberine chloride, (b) trans-resveratrol, and (c) ellagic acid with active site of target protein EGFR.

Based on scoring values/binding capacity, docking results of the top three naturally occurring compounds (berberine chloride, trans-resveratrol, and ellagic acid) are mentioned in Table 6, while their 3D interactions with active sites of EGFR protein are presented in Figures 6(a)–6(c). Based on scoring value, berberine chloride had the highest binding potential (SV = −24.30 kcal/mol) with EGFR protein, followed by trans-resveratrol and ellagic acid. Relatively, the scoring values of these compounds were even lower than the scoring values of HMs with the same protein. Berberine chloride depicted pi-sulfur, alkyl, and pi-alkyl interactions with active residues (Met790, Leu777, Met766) of the target protein (Figure 6(a)). Likewise, trans-resveratrol showed conventional hydrogen, pi-sigma, pi-pi t-shaped, and pi-sulfur interactions with active residues (Phe856, Lys745, Met790) of target protein (Figure 6(b)), and ellagic acid exhibited conventional hydrogen, pi-lone pair, and pi-pi t-shaped interactions with residues (Phe856, Lys745, Leu788) of target protein (Figure 6(c)).

Our findings indicate that natural drug candidates have significant potential to inhibit the binding capacity of HMs with EGFR protein. As, the interaction energies between natural compounds and EGFR protein, was less than that of heavy metals, which means that its binding affinity to form a complex is substantial (Table 6). Therefore, the aforementioned natural compounds could be an appropriate option to prevent lung cancer. Furthermore, root and stem bark of “Goldenseal, grapes, turmeric, and barberry” are rich in “berberine chloride,” while “Trans-resveratrol” a polyphenolic compound present in “grape” and “Ellagic acid” present in “strawberries, blackberries grapes, walnuts and nuts” [51–53]. Consequently, daily intake of these fruits and medicinal plants could an alternative therapy that may play important role in the prevention of lung cancer, specifically caused by heavy metals' toxicity.

**Figure 7:**
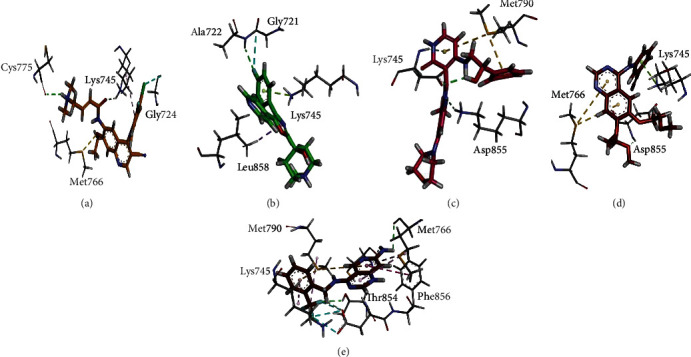
3D interactions of synthetic drug candidates (a) ZINC00602803, (b) ZINC00602685, (c) ZINC06718468, (d) ZINC01546066, and (e) ZINC13743457 with binding site of target protein EGFR.

Docking results of top five synthetic compounds viz., IDs ZINC00602803, ZINC00602685, ZINC06718468, ZINC01546066, and ZINC13743457 are mentioned in Table 6. Comparatively, the binding capacity of all these compounds with target protein was higher than HMs, because of their low scoring values. Furthermore, the binding affinity of abovementioned synthetic compounds with EGFR protein was confirmed by a 3D interaction plot (Figures 7(a)–7(e)). As shown in 3D networks, synthetic compounds bind to the active pocket of the targeted protein, and ligand atoms showed sidechain acceptor, sidechain donor, backbone acceptor, and backbone donor interactions with acidic, basic, greasy, and polar residues of receptor atoms.

As shown in Figure 7(a), ZINC00602803 had conventional hydrogen, halogen, and pi-sulfur bonds with the active residues (Cys775, Lys745, Gly724, Met766) of the target protein. Likewise, “ZINC00602685” had conventional hydrogen, pi-lone pair, pi-sigma, and halogen interactions with the active residues (Ala722, Gly721, Lys745, Leu858) of target protein (Figure 7(b)), whereas “ZINC06718468” showed conventional hydrogen, pi-sulfur, and pi-donor hydrogen bonding with active residues (Lys745, Met790, Asp855) of target protein (Figure 7(c)), and “ZINC01546066” showed conventional hydrogen, pi-sulfur, and pi-lone pair interactions with the active residues (Met766, Lys745, Asp855) of target protein (Figure 7(d)), and “ZINC13743457” compound depicted conventional hydrogen, halogen, pi-sulfur, and pi-pi t-shaped interactions with active residues (Met790, Lys745, Thr854, Phe856, Met766) of target protein (Figure 7(e)). Comparatively, three synthetic drug candidates, i.e., ZINC00602803, ZINC00602685, and ZINC06718468, showed a highly significant association with target protein based on the lowest scoring values (-30.99, -29.75, and -29.68 kcal/mol, respectively). It was noted that the binding affinity of ZINC00602803 was higher than all HMs and even that of standard lung cancer drugs.

## 4. Conclusions

*In silico* assessment of molecular interaction confirms the association of heavy metals with the oncoprotein EGFR. Our findings revealed that bioaccumulation of heavy metals in human and animal bodies may involve lung cancer along with other serious health disorders. So, drug validation analysis indicates that both natural and synthetic compounds have a strong binding affinity with EGFR protein which could inhibit the active site of EGFR against heavy metal exposure. As, the binding affinity of berberine chloride, and synthetic compound (ZINC0060280), with the target protein, was even higher than the standard drugs used as control. Therefore, these compounds could be a more appropriate and safe option for the treatment and prevention of lung cancer, specifically caused by heavy metal toxicity. However, we suggest *in vitro* and *in vivo* validation of natural and synthetic compounds specifically that showed significant associations with EGFR protein.

## Figures and Tables

**Table 1:** Scoring values of protein coding NSCLC genes.

S. #	Symbol	Description	Score
1	EGFR	Epidermal growth factor receptor	21.25
2	KRAS	KRAS proto-oncogene, GTPase	14.42
3	ALK	ALK receptor tyrosine kinase	12.58
4	ERBB2	Erb-B2 receptor tyrosine kinase 2	12.47

**Table 2:** Cross-docking results for various PDB IDs from EGFR.

PDB IDS of EGFR protein	RMSD (Å)		
	6DUK	5GTY	3IKA
6DUK	0.89	0.98	**1.73**
5GTY	1.01	1.10	***2.73***
3IKA	***2.13***	***2.24***	**1.67**

**Table 3:** Properties of heavy metals' compounds.

S. #	Metal	MW (mg/mol)	WS (mol/L)	Signal	AOTC
1	Arsenic	74.92	0.463	Danger	3
2	Lead	207.0	0.046	Danger	3
3	Cadmium	112.4	1.090	Danger	3
4	Mercury	200.6	0.064	Danger	3
5	Nickel	58.69	7.180	Danger	3
6	Thallium	204.4	—	Danger	3
7	Copper	63.55	6.620	Danger	3
8	Antimony	121.8	—	Danger	3
9	Bismuth	209.0	0.043	Warning	3
10	Cerium	140.1	0.381	Warning	3
11	Chromium	52.00	1.670	Danger	3
12	Gallium	69.72	1.240	Danger	3
13	Gold	197.0	0.054	—	3
14	Platinum	195.1	0.031	Danger	3
15	Tellurium	127.6	0.469	Danger	3
16	Silver	107.9	0.653	Warning	3
17	Tin	118.7	—	Warning	3
18	Uranium	238.0	0.074	Danger	3
19	Vanadium	50.94	1.700	—	3
20	Cobalt	59.93	1.480	Danger	3
21	Manganese	54.94	1.590	Warning	3
22	Iron	55.85	11.30	Danger	3
23	Zinc	65.38	5.260	Danger	3

MW: molecular weight; WS: water solubility; Sig: signal; AOTC: acute oral toxicity class.

**Table 4:** Molecular docking results of heavy metals' compounds with EGFR protein.

S. #	MC	SV	RMSD	E-conf.
1	Tinidazole	-20.42	1.455	41.16
2	Thallium, bromodimethyl	-7.865	1.467	-18.09
3	Silver acetate	-7.748	2.361	-42.74
4	Mercuric cyanide	-6.323	3.830	-291.7
5	Arsenate	-5.700	1.149	-154.4
6	Tellurium hexafluoride	-5.396	0.570	-1139
7	Lead sulfide	-4.744	1.157	-71.08
8	Antimony trichloride	-4.256	1.131	-17.03
9	Vanadium tetrachloride	-4.209	1.266	-83.48
10	Chromium iodide	-4.109	1.414	-34.01
11	Bismuth trichloride	-3.693	0.563	-15.09
12	Platinum tetrachloride	-3.462	1.341	-36.47
13	Gold bromide	-3.339	1.369	-3.975
14	Manganese dioxide	-3.156	1.715	-120.8
15	Ferric chloride	-2.972	2.541	-23.12
16	Nickel chloride	-2.971	1.105	-39.53
17	Uranium trioxide	-2.951	0.733	-253.5
18	Cerium disulfide	-2.758	4.094	-61.50
19	Cobalt (II) bromide	-2.708	1.443	-64.85
20	Zinc diflouride	-2.007	1.256	-11.85
21	Gallium iodide	-1.951	0.935	-26.38
22	Cupric chloride	-1.825	2.994	-12.83
23	Cadmium bromide	-1.582	1.133	-11.94

MC: metal compounds; SV: scoring values (kcal/mol); RMSD values: root means square deviation (Å); E-conf: expected confirmation.

**Table 5:** Predicted statistical parameters for each drug-drug interaction network.

Networks	AD	AWD	ND	GD	M	APL	Nn	Ne
1	2.361	2.847	1	0.017	0.619	1	72	085
2	2.899	5.217	1	0.007	0.589	1	207	300
3	2.797	5.359	1	0.005	0.602	1	276	386
4	2.634	5.935	1	0.011	0.579	1	123	162
5	2.544	5.645	1	0.008	0.629	1	169	215
6	2.583	6.953	1	0.008	0.618	1	169	218
7	2.626	4.746	1	0.012	0.621	1	131	172
8	2.808	4.739	1	0.005	0.585	1	287	403
SIN	3.566	1.925	1	0.004	0.518	1	415	740

AD: average degree; AWD: average weighted degree; ND: network diameter; GD: graph density; M: modularity; APL: average path length; Nn: number of nodes; Ne: number of edges; SIN: strongly interacted network.

**Table 6:** Docking results of the controls, natural, and synthetic compounds with EGFR protein.

S.#	Name/formula/ZINC-ID	Structure	SV values	RMSD
Controls				
1	Erlotinib (C_22_H_23_N_3_O_4_)	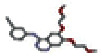	−29.49 ± 1.3	1.87
2	Gefitinib (C_22_H_24_ClFN_4_O_3_)	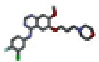	−29.33 ± 0.9	1.19
3	Afatinib (C_24_H_2_5ClFN_5_O_3_)	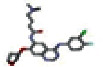	−29.99 ± 0.6	1.23
4	Native cocrystallized ligand	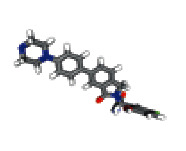	−31.64 ± 1.1	1.42
Natural compounds				
1	ZINC03779067 (berberine chloride-C_20_H_22_ClNO_6_)	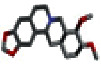	−24.30 ± 0.8	1.05
2	ZINC12353732 (trans-resveratrol-C_14_H_12_O_3_)	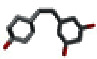	−20.37 ± 0.5	1.60
3	ZINC03872446 (ellagic acid-C_14_H_6_O_8_)	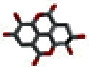	−18.63 ± 0.7	0.80
Synthetic compounds				
1	ZINC00602803	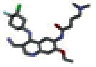	−30.99 ± 0.7	1.30
2	ZINC00602685	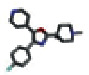	−29.75 ± 0.3	1.08
3	ZINC06718468	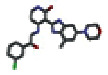	−29.68 ± 1.2	1.59
4	ZINC01546066	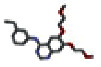	−29.08 ± 1.3	1.60
5	ZINC13743457	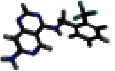	−22.69 ± 0.1	0.59

SV: scoring values (kcal/mol); RMSD values: root means square deviation (Å); SD value: standard deviation value.

## Data Availability

All data is provided in this article; however, for any information, corresponding author may be consulted.
